# A Practical Treatise on the Diseases of the Scalp

**Published:** 1843-01

**Authors:** 


### Art. IX.

*A Practical Treatise on the Diseases of the Scalp.* By John E. Erichsen, Member of the Royal College of Surgeons, London.—*London*, 1842. 8vo, pp. 192.

At first sight it may appear strange that the knowledge of cutaneous diseases (which from their situation come so immediately under the eye,) has not kept pace with that of other and apparently much more obscure disorders; and that the information possessed by the great mass of practitioners respecting them, should be more slender and superficial than upon almost any other class of diseases; and we may perhaps be excused for noticing some of the circumstances which appear to have contributed to this lack of knowledge.

The great number of these diseases would not be an obstacle to our knowledge of them, if their characters were constant, and their appearance in the different stages always similar; this, however, is far from being the case; many cutaneous diseases at the time when they come under the notice of the practitioner being much altered in appearance, partly from neglect on the patient's side, partly from the chronic nature of many of these affections, and often from the abuse of a variety of local applications. Many of the diseases of the skin, too, are not of that importance to induce the patient to seek admission into hospitals. The same facilities are not afforded for the study of cutaneous diseases in this as in other countries. We possess no public institution at all approaching the hospital of St. Louis, for the reception and treatment of these affections, where the various forms of the same disease, whether arising from difference in situation or other cause are grouped together, and can be exhibited to pupils. The confusion created by the difference in nomenclature and classification adopted by writers in this country and on the continent, (the same diseases in different stages being sometimes described as dissimilar affections, and the same terms employed in quite a different sense,) has also operated in retarding the knowledge of these diseases. But, there is another and perhaps a more powerful cause still to be noticed. Many of these cutaneous affections being the ordinary attendants upon poverty and want of cleanliness, are necessarily in a great measure confined to the lower classes of society: hence there is not the same encouragement to their study, which that of many more fashionable diseases presents; and the practitioner who devotes himself to their investigation and acquires character in their treatment, will seldom arrive at fortune, though he may at fame; their study has consequently never been very popular, and but few men of talent (comparatively speaking,) have devoted themselves to this branch of pathology. This perhaps applies more particularly to the cutaneous affections of the scalp than of the body generally; for though these diseases are not confined to the lower orders, they are much more frequent among them than the upper classes; and if they do occur among the latter, instead of being confided to the regular practitioner, they are usually handed over to some old woman, or equally ignorant quack. We trust, however, the day is not distant when a change will take place in this respect, when the ignorance of the true nature and consequently of the proper scientific treatment of these diseases will be no longer general; and we believe the treatise of Mr. Erichsen is materially calculated to bring about such a result.

Chap. I is devoted to an outline of the classification adopted, and to the causes, prognosis, diagnosis, and treatment generally of scalp diseases. After some preliminary observations upon the neglect of their study, and upon the confusion which has resulted from grouping together under the terms *porrigo*, and *tinea capitis*, diseases differing totally from one another, Mr. Erichsen arranges the cutaneous diseases which ordinarily affect the scalp in four classes, viz., Vesicular, Pustular, Tubercular, and Scaly. The vesicular diseases belong to the genera *eczema* and *herpes;* the pustular to the genus *impetigo;* the tubercular to *favus*, and the scaly to the genus *pityriasis.*

Eczema. The varieties of eczema are among the most common cutaneous affections of the scalp. With the exception of impetigo, says Mr. Erichsen, there is no disease which affects the scalp more frequently, and Mr. Phillips, in his lectures in the present volume of the Medical Gazette, observes that a very large proportion of the examples of scalp disease which come under his observation at the St. Marlylebone infirmary, are cases of this affection.

Willan and Bateman make three divisions of eczema, viz., E. solare, E. impetigenodes, and E. rubrum. Mr. Erichsen follows the more scientific arrangement of Biett and Rayer in dividing the disease into acute and chronic eczema.

*Acute Eczema* is more frequently seen upon other parts of the body than on the scalp, as the organs of generation, groin, axilla, ears, back of the neck. A remarkable variety of this affection arising from the irritation of mercury is familiar to surgeons. Acute eczema of the ears is thus described by Mr. Erichsen:

"The ears become exceedingly red, tense, hot, and shining; a number of small vesicles then appear, which contain a clear serum, of a reddish or yellowish colour; when these give way, the fluid which is effused forms thin scales, or scabs, which are cracked in all directions. The pinna attains a very large size, becoming hypertrophied and often fissured; sometimes indeed the swelling takes place to such an extent as to block up the meatus auditorius externus, giving rise to temporary deafness." (p. 42.)

The chronic forms of eczema of the scalp come more frequently under the eye of the practitioner than the acute; and while little difficulty is experienced in the diagnosis of the latter, the former may be very readily confounded with several other cutaneous affections of this part.

Mr. Erichsen makes three divisions of chronic eczema of the scalp; "1st, simple chronic eczema, which may be either moist or dry; 2d, eczema furfuracea, corresponding to the porrigo furfurosa of Willan, tinea furfuracea of others, and the achor lactuminosus of Alibert; 3d, eczema amiantacea, the teigne amiantacée or porrigo asbestina of Alibert, who was the first to describe it."

*Simple chronic eczema* is either moist or dry, and these two varieties frequently pass into one another, the moist becoming dry as the disease tends to get well. In the moist variety "the hair looks as if it had been soaked in a thin solution of gum-arabic, being matted together in locks, which have a dirty yellowish-gray moist appearance, and between and under which the inflamed scalp may be seen to be perforated by a number of minute openings, which pour forth the discharge; as it lessens in quantity soft yellowish-gray scabs form, which gradually lose their moist appearance. A smell resembling that of the fumes of acetic acid is often evolved from it." The dry variety is characterized by scales or scabs of a yellowish-white or yellowish-green colour, usually lamellated, a line or two in thickness, separated from one another by cracks or fissures, at the bottom of which the inflamed scalp is seen covered by a mealy powder, the detritus of the scabs.

These two varieties of eczema often pass insensibly into one another; both are common in infants at the breast, and during the second dentition: and children with light hair, fair complexion, and scrofulous constitution, according to our author and Rayer, appear to be more frequently the subject of it, than those of an opposite complexion; though Mr. Phillips says he finds it to be very common also in children of a dark complexion; simple chronic eczema of the scalp is much more frequent among the lower than the upper classes; it often spreads over a large portion of the scalp, and extends to the forehead, face, ears, or back of neck; it does not appear to be a contagious disease; its duration varies according to the treatment pursued, if judicious from three to six weeks; if the disease is neglected it may persist for many months; in our experience it is always more tedious in the adult than in the infant. The older writers imagined that the eruption of eczema in children teething, protected them in some measure from attacks of convulsions or diarrhœa.

*Diagnosis.* The only cutaneous affection of the scalp with which this form of eczema is very liable to be confounded is impetigo eczematosa.

"In the later stages of the disease the scabs of eczema will be observed to be of a lighter colour, being yellowish-white or yellowish-gray, and more lamellated than those of impetigo, which are thicker, of an irregular shape, and of a yellowish-brown colour, presenting somewhat of a varnished or glazed appearance, and having a tendency to agglomerate in masses, whilst those of eczema appear rather to be spread out in layers, and are more friable and opaque." (p. 52.)

*Treatment.* The treatment of acute eczema of the scalp is simple and usually sufficiently obvious; that of chronic eczema on the contrary is frequently tedious. The hair should be cut close, shaving it is not necessary; poultices and fomentations are only employed by Mr. E. in the first instance to detach the scabs, afterwards he recommends (in the dry chronic form) lotions containing either the pure alkalies or their carbonates and sulphurets, in the proportion of one to three drachms to the pint of water. In the moist form ointments containing nitrate of silver, iodide of sulphur, bichloride, nitrate, or ammoniochloride of mercury, or sulphate of zinc are often necessary; the ointment should be applied at night and washed off in the morning with any of the lotions before mentioned. We are surprised not to find any mention made of creosote, as a local application in the several forms of chronic eczema of the scalp; we have often derived much benefit from this substance, both in the form of lotion and ointment; nothing relieves the pruritus more effectually or more quickly than it. The oil-skin cap, in these cases, we entirely agree with the author in disapproving of; a clean linen cap is decidedly preferable. The constitutional treatment must vary according to several circumstances; we believe, however, that in the majority of cases it is at least of equal importance with the local.

We must pass over the author's observations upon eczema furfuracea, and E. amiantacea. In concluding a short account of the treatment of those two forms of the disease he justly observes, that "they will be found under any mode of treatment to be exceedingly rebellious and difficult of cure, frequently persisting for a great length of time under most judiciously directed means; and appearing at length to cease spontaneously, rather than to yield to art."

The only other vesicular diseases which occur upon the scalp, are herpes circinnatus and H. zoster, the latter very rarely. The former is sometimes termed vesicular ring-worm; both these affections, however, are uncommon upon the scalp, and both are described with sufficient accuracy in most systematic treatises upon cutaneous diseases.

IMPETIGO. The only pustular disease which (according to Mr. Erichsen) commonly affects the scalp is impetigo; and two distinct forms of pustule are met with in this affection, viz., *psydracia* and *achores.*

"The psydracium is a pustule that is usually small and irregularly circumscribed, without much inflammation about the base, terminating in scabs which vary considerably in size and figure. It characterizes impetigo sparsa. The achores are larger, more superficial and confluent, with a more extensive inflammation about the base; when they give way, yellow or brown-coloured scabs result, which however are not so thick or so dark as those that characterize the psydracia. This form of pustule occurs in impetigo eczematosa and impetigo granulata." (p. 78.)

Impetigo of the scalp, in our author's arrangement, comprises three species, viz., impetigo sparsa (porrigo favosa, Willan), impetigo granulata (tinea, or porrigo granulata), and impetigo eczematosa (porrigo, or impetigo larvalis).

*Impetigo sparsa.* We cannot help remarking that the name which Mr. Erichsen has chosen for this affection of the scalp appears to be rather an unfortunate one. The term impetigo sparsa had been already applied by Bateman, Biett, and others, to a pustular disease which particularly affects the lower extremities, and is but rarely seen upon the scalp. The porrigo favosa of Cazenave and Schedel is the porrigo lupinosa of Willan, and the favus dispersus of our author, a contagious disease. Mr. Phillips (Lectures, Medical Gazette, 1842,) considers the porrigo granulata of Willan as identical with the impetigo sparsa of other parts; and Burns looks upon the porrigo favosa and porrigo larvalis as being identical. "The small pustules of porrigo favosa (observes Rayer, Treatise on the Diseases of the Skin, translated by Dickinson, p. 147,) are never humid like those of impetigo, they are covered by a yellow dry crust of a cup shape. It is more difficult (he adds) to establish a clear line of demarcation between the pustules of porrigo larvalis and those of impetigo sparsa; some pathologists maintain that these are two distinct diseases, and others suppose that the difference between them results from the different texture of the skin of the regions on which they are developed, and the not less remarkable difference in the ages of the subjects of attack." Hence we fear that the author's application of this term will, in the present instance, tend rather to increase than to do away with the confusion which already exists in the nomenclature of these diseases.

Mr. Erichsen's description of impetigo granulata and impetigo eczematosa appears to be very accurate and complete; but as these diseases, under the names porrigo granulata and porrigo larvalis, are noticed at considerable length in works already in the hands of the profession, we shall not expatiate here upon their characters; merely observing that Bateman had already suggested the name impetigo larvalis, for the disease which Willan named porrigo larvalis; and although impetigo eczematosa is perhaps a better name, less confusion would probably have resulted from adopting his suggestion.

*Causes of impetigo of the scalp.* Age appears to have some influence upon the form which the disease assumes, impetigo eczematosa being most common about the period of the first dentition, and I. sparsa and I. granulata about the second dentition. I. eczematosa and I. sparsa are most frequently seen in children with fair skin, light ruddy complexion, and "who are frequently remarkable for their beauty." I. granulata, on the other hand, occurs in children with dark complexion, hair, and eyes, and a spare conformation. The varieties of this disease occur both in weakly children, who are badly fed and clothed, and in healthy children, who are over-fed. In many cases the affection cannot be referred to any cause. None of the species, according to Mr. E., are contagious; Alibert, however, notices the case of an infant who was inoculated with the impetigo eczematosa (porrigo larvalis), and took it.

*Prognosis.* "Impetigo of the scalp," Mr. Erichsen observes, "can scarcely ever be said to be a dangerous disease." Dr. Dewes, however

(in his Treatise on the Diseases of Children), states that he has seen two instances of death from porrigo larvalis (impetigo eczematosa). In these cases the itching was incessant, the disease continued many months, and the children were carried off by profuse diarrhœa. Mr. Plumbe (in his Practical Treatise on the Diseases of the Scalp) also notices the fact of this disease sometimes ending in marasmus, diarrhœa, and fatal hectic. On the other hand, Underwood (in his Treatise on the Diseases of Children,) says, he "never saw an infant much loaded with the porrigo larvalis (impetigo eczematosa) but it has always been healthy, and cut its teeth remarkably well; in general, however, although it appears in the most healthy children, yet it is the consequence of repletion, and the irritation of undigested food upon highly excitable systems; and in these it probably prevents the attacks of more formidable diseases." The duration of impetigo of the scalp is from a few weeks to several months, or even longer; I. granulata is the most tedious; it is, however, the least common species; the disease is liable to recur a second or third time, after its apparent cure in the first instance; it does not destroy the bulbs of the hair, and leaves neither baldness nor cicatrices behind. It is not limited to the scalp. I. eczematosa often affects simultaneously the face, and scalp, and may spread to the neck, ears, and shoulders; it is sometimes complicated with inflammation of the conjunctiva, and mucous membrane of the meatus auditorius externus; indeed Mr. Christian, of the Liverpool Ophthalmic Infirmary, has described an inflammation of the eye, so common an attendant upon this affection, that he named it porriginous ophthalmia.

*Treatment.* When impetigo occurs in infants during the first dentition, and in a mild form, Mr. E. says, "it becomes a question whether we should not rather leave it to nature, attending merely to cleanliness, and making use of such simple palliatives as may be necessary to lessen irritation, than employ any very active means for its cure." This more particularly applies to impetigo eczematosa. Plenck considered it dangerous to check suddenly the eruption, by external applications, imagining that it was likely to produce ophthalmia, cough, anasarca, hydrocephalus, &c. Dr. Granville thinks he has also observed this to be the case; and Billard (as quoted by Mr. E.) says he has always found that those children who were allowed to get well of this affection slowly and naturally, had a fine clear complexion, and were remarkable for their healthy appearance; and that, on the contrary, the sudden cure of it was very frequently attended by evident derangement of the health of the child. "In some rare cases (observe Cazenave and Schedel, p. 220,) the appearance of porrigo larvalis (impetigo eczematosa) has seemed to have acted beneficially, and to have tended to the cure of more dangerous diseases." "In the generality of cases (according to Biett, American edit. p. 219), lotions of tepid water, milk, or decoction of mallows, which unite the double advantage of preventing the scabs from accumulating, and of calming the violence of the inflammation, should constitute the whole treatment; and, in children at the breast, the best remedy is to direct the nurse to wash the diseased parts with her milk."

When, however, the disease occurs in older children, and is attended by greater constitutional disturbance, more active measures must be resorted to. It is seldom necessary to shave the head, clipping the hair close over and around the diseased parts will generally be sufficient; and the parts should be afterwards covered with an emollient cataplasm of linseed meal, or bread and milk; if the irritation is so great as to deprive the child of sleep, a few leeches behind the ears, or below the angles of the jaws, will, as recommended by Rayer and our author, be of service; general bloodletting is never necessary, and all stimulating applications to the head are injurious. The general treatment of this stage consists in keeping the bowels open by mild laxatives, such as "sulphate of magnesia, tartrate of potash, sulphate of soda, with a few grains of the hydrarg. cum creta, or calomel." We entirely agree with Mr. E. in the following remarks:

"In the acute stage of impetigo of the scalp all specific remedies are perfectly useless, and indeed are positively injurious; and it cannot be impressed too strongly that the only mode of treatment, whether topical or general, that is likely to benefit the patient, is one conducted upon mild, antiphlogistic principles." (p. 100.)

When the disease has lasted for a considerable time, and the inflammatory symptoms have been perfectly subdued, we may then, after the removal of the scabs, have recourse to stimulating topical applications, and Mr. E. finds none so useful as a lotion composed of sulphuret of potassium (potash?) in the proportion of one to three drachms to the pint of water, and applied several times a day; at the same time, the Harrowgate waters, or other sulphureous waters, natural or artificial, may be administered internally. Cazenave and Schedel prefer a lotion composed of one drachm of sulphuret of potash, and two drachms of subcarbonate of potash or soda, to the pint of water. Mr. Phillips recommends, after the application of the lotion, the parts to be covered with the common sulphur ointment, which is to be well washed off with soft or yellow soap, morning and evening, before the lotion is reapplied; in some instances Mr. E. employs either an ointment of the nitrate of mercury, or of the peroxide of the metal; but, in general, he considers that every useful purpose will be answered by lotions. Dr. A. T. Thomson, in his notes to Bateman's work, says he has seen no ointment so generally beneficial in porrigo larvalis (I. eczematosa), as the ung. hyd. nit. diluted with seven parts of spermaceti ointment. Rayer approves of sulphureous lotions, or ointments of the nitrate of mercury. Lotions composed of bichloride of mercury, alum, sulphate of copper, nitrate of silver, &c., have also been recommended, but Mr. E. considers them all inferior to the sulphuret. When the itching is very troublesome, he employs a lotion composed of oxide of zinc, suspended in rose-water, with the addition of a few drops of hydrocyanic acid.

While these local measures are being employed, the general health must be attended to; indeed Dr. Crampton (in vol. iv. of the Transactions of the Association of the King and Queen's College of Physicians,) has given a series of cases of this and other forms of scalp affections, treated almost altogether by constitutional remedies, of which the chief were purgatives and the warm bath. Among constitutional remedies, the diluted mineral acids have been, in our hands, more efficacious than any other medicines. Mr. E. prefers the nitric or the nitro-muriatic; we generally employ the dilute sulphuric of the Pharmacopœia. Mr. E. says he has never met with any cases of impetigo of the scalp that did not yield to the judicious employment of the above plan of treatment, or that necessitated the use of arsenical preparations, or of the remedies that are usually looked upon as specifics in diseases of the skin. No doubt, in the great majority of cases, the foregoing remedies are perfectly adequate to the cure of each and every form of impetigo of the scalp, but cases occasionally occur where they fail, and more energetic measures are required. In such, the bichloride of mercury, in the dose of one sixteenth of a grain, or Fowler's solution of arsenic, in minute doses, will be generally productive of benefit. In vol. viii. of the Philadelphia Journal of the Medical Sciences, Dr. Hendrie has given some cases of impetigo which proved very obstinate, and were cured by the expressed juice of the sanguinaria canadensis.

Favus. Under the genuine name favus, which corresponds to the porrigo of Willan, Bateman, and others; to the teigne of Alibert and Mahon, and to the tinea of other writers, Mr. Erichsen includes only two species, viz., *favus dispersus* (porrigo lupinosa, Willan,) and *favus confertus* (porrigo scutulata of others). Hitherto the greatest confusion has prevailed in this division of the subject. Thus Willan and Bateman make six species of porrigo, viz., P. larvalis, P. furfurans, P. lupinosa, P. scutulata, P. favosa, and P. decalvans. Mr. E., however, has shown that porrigo larvalis and P. favosa are species of impetigo, P. furfurans a species of eczema; and that the partial baldness described by Willan under the name P. decalvans, is merely the result of the destruction of the hair from a previous attack of favus dispersus, or confertus. Alibert has described five varieties, under the genuine name tinea, viz., teigne faveuse, T. granulée, T. furfuracée, T. muqueuse, and T. amiantacée. The teigne faveuse of Alibert is very distinct from the porrigo favosa of Willan, and the T. furfuracée and T. amiantacée are varieties of chronic eczema. Cazenave and Schedel make four species of porrigo; viz., P. favosa, P. scutulata, P. granulata, and P. larvalis; the P. granulata and P. larvalis they consider to be more closely allied to impetigo than to porrigo; and their P. favosa is identical with the P. lupinosa of Willan. Rayer admits four species of tinea (which term he employs only because it has been long in use), viz., T. favosa, T. annulare, T. granulata, and T. mucosa. He, however, regards each as perfectly distinct, and not as species or varieties of the same disease. M. Mahon, in his work on the subject, makes eight species of tinea, or teigne, but, under this general term, he has grouped together vesicular, pustular, tubercular, and scaly diseases. In confining the genus *favus* to two well-marked species, Mr. E. but follows out an idea long since suggested by Biett, in his lectures. "If species must be made (MM. Cazenave and Schedel observe, p. 233, ed. 1828), M. Biett is of opinion that they must be reduced to two, P. favosa and P. scutulata (favus dispersus and F. confertus of Mr. E.); in fact these two species alone present characters which are not to be found in other orders."

*Seat of favus.* Respecting the seat of favus much contrariety of opinion has existed; our author coincides with Rayer in considering favous matter to be originally deposited in the dilated cavities of the cuticular conduits of the hair; in other words, within the hair follicles, but upon the surface of the prolongation of the cuticle which lines them.

*Nature of favus.* The majority of writers consider favus to be a pustular disease; Mahon regards it as a morbid secretion of the sebaceous follicles (in which he places its seat), and recently it has been supposed to be a parasitical growth, analogous to fungi, or to those vegetable productions which form in some fermented liquors. Mr. E. considers favous matter to be a modification of *tubercle.* By the term tubercular he does not mean "a disease like lupus, characterized by small, firm tumours, but one the essential nature of which consists in the deposition of that heterologous formation called tubercle." Into the arguments brought forward by our author in proof of his position, want of space forbids us to enter; the following summary contains the substance of them.

"Favus, in its elementary form, differs from pustule,—1st, in the favous matter being poured out upon a free surface, and not upon or within the cutis, and under the cuticle:—2d, the favous tubercle is frequently chronic, existing in an imperfectly developed state for a length of time, which is never the case with pustule. Favous matter differs from pus,—1st, in concreting very quickly after it is poured out, even before exposure to the air:—2d, in its chemical composition; favous matter containing much more earthy salts, and coagulated albumen, than pus. Favus and tubercle agree in their seat [the lining membrane of the hair-follicles appearing to Mr. E. to partake, in a great measure, of the characters of mucous tissue], in the manner of their formation (both being eliminated in a fluid state, but solidifying very quickly,) in their mode of growth (by eccentric deposition, and not by any increase from within,) in form, colour, and chemical composition; in most of the causes that predipose to, or excite them; in the age at which they most frequently occur; and in the colour of the cicatrices which are left." (p. 117.)

*Diagnosis.* Favus being the only really contagious disease of the scalp, its diagnosis is of the utmost importance, and, in the majority of cases, this presents little difficulty. "It is distinguished from all other cutaneous affections of the scalp, by its small, yellow tubercles; by its circular, dry, cup-shaped crusts; by the baldness that it occasions; and by its contagious nature."

*Treatment.* There are probably few diseases (as Mr. E. remarks) for the cure of which so many different modes of treatment have been employed, and such a variety of specific remedies vaunted as for favus; and this (he adds) can be readily accounted for, when we reflect upon the obscurity which has hung over its diagnosis, and on the extreme obstinacy with which (when fully established) it resists almost every plan of treatment.

In the local treatment there are three indications to be fulfilled, in which almost all writers on the disease are agreed,—viz., to detach the scabs, to remove the hair from the diseased follicles, and to set up a new action in the affected part.

In order to detach the crusts and scabs, the hair must be cut short; and poultices either of bread and milk, linseed, or oatmeal applied; in very obstinate cases we may have recourse to brown soft soap, reduced to a stiff jelly, or soap and oatmeal half boiled, and mixed as recommended by Dr. Crampton. At the same time a lotion composed of an ounce of diluted hydrochloric acid to the pint of water may be employed; and the head must be regularly washed with soap and water.

The second indication consists in the removal of the hair from the diseased follicles, which, according to Rayer, is as essential in the treatment of favus as is the removal of the nail in some forms of onychia. Biett, however, questions this assertion. "Is the presence of the hair

(he asks) as prejudicial as some authors insist? and when it falls off from the effects of the disease does the latter disappear? On the contrary the scabs often remain for years on places where there is no hair." To effect the removal of the hair an adhesive plaster, (called the pitchcap,) composed of black pitch, resin, and Burgundy pitch was formerly applied tightly to the head after the scabs had been removed by poultices and the hair had been shaved; at the end of some days it was suddenly raised in a contrary direction to that in which the hair grows; and a second was applied; the process was repeated every third or fourth day for months. The cap has now been abandoned many years by regular practitioners, "and we can scarcely credit (as Biett observes,) how ignorance could dare to make use of so cruel a method." Mr. Plumbe employed a forceps for extracting the hairs, and this plan, though very tedious, had the advantage over the 'pitch-cap,' of being much less painful, and of only removing the hairs from the diseased parts; it is however seldom or never employed at the present day. The hair may be readily detached by mild depilatories with certainty and without pain; for this purpose Biett employed an ointment, composed of sub-carbonate of potash or soda and lard, in the proportion of one or two drachms to the ounce, and rubbed on the diseased parts for five or ten minutes each day; mild alkaline lotions, composed of two drachms of those salts to a pint of water being employed at the same time.

For the fulfilment of the third indication, that of setting up a new action in the part, Mr. Erichsen gives the preference to the iodide of sulphur, sulphuret of potassium, (potash?) and carbonate of potash. The iodide of sulphur is employed in the form of ointment made with ten or twenty grains to the ounce of lard. The carbonate of potash lotion has been already mentioned; and the sulphuret of potash lotion is used to remove the scurfy condition of the scalp left after the separation of the scabs.

Mr. Erichsen's observations upon the constitutional treatment of favus are few and unimportant; he evidently has no confidence in it alone, but trusts chiefly to local remedies. In concluding his account of favus he makes the following remarks:

> "It may be confidently stated, that by patience and perseverance in the employment of the means above indicated, and by scrupulous attention to cleanliness there are few cases of favus that will not speedily be cured; and that this disease, instead of continuing as it ever has been an opprobrium to medical men, can be as successfully treated, in the great majority of cases, as most other cutaneous affections." (p. 160.)

Chapter V. of Mr. Erichsen's work is devoted to the scaly diseases only one of which, pityriasis, ordinarily affects the scalp; chapter VI. to the diagnosis of the diseases of the scalp; and chapter VII. to the diseases of the hair. But as we have already exceeded the limits assigned to this article we must conclude. We cannot do so, however, without expressing our approbation of the beauty and fidelity of the plates, which considerably enhance the value and utility of the work; and we have no hesitation in expressing our belief that the publication of Mr. Erichsen's work will give an impulse to the study of the cutaneous affections of the scalp, that was much wanting. We would earnestly recommend its perusal to all who desire to treat those diseases upon scientific, rather than empirical principles.

